# Coupled Analysis of In Vitro and Histology Tissue Samples to Quantify Structure-Function Relationship

**DOI:** 10.1371/journal.pone.0032227

**Published:** 2012-03-30

**Authors:** Evrim Acar, George E. Plopper, Bülent Yener

**Affiliations:** 1 Faculty of Life Sciences, University of Copenhagen, Frederiksberg C, Denmark; 2 Department of Biology, Rensselaer Polytechnic Institute, Troy, New York, United States of America; 3 Department of Computer Science, Rensselaer Polytechnic Institute, Troy, New York, United States of America; Medical University of Graz, Austria

## Abstract

The *structure/function relationship* is fundamental to our understanding of biological systems at all levels, and drives most, if not all, techniques for detecting, diagnosing, and treating disease. However, at the tissue level of biological complexity we encounter a gap in the structure/function relationship: having accumulated an extraordinary amount of detailed information about biological tissues at the cellular and subcellular level, we cannot assemble it in a way that explains the correspondingly complex biological functions these structures perform. To help close this information gap we define here several quantitative temperospatial features that link tissue structure to its corresponding biological function. Both histological images of human tissue samples and fluorescence images of three-dimensional cultures of human cells are used to compare the accuracy of in vitro culture models with their corresponding human tissues. To the best of our knowledge, there is no prior work on a quantitative comparison of histology and in vitro samples. Features are calculated from graph theoretical representations of tissue structures and the data are analyzed in the form of matrices and higher-order tensors using matrix and tensor factorization methods, with a goal of differentiating between cancerous and healthy states of brain, breast, and bone tissues. We also show that our techniques can differentiate between the structural organization of native tissues and their corresponding in vitro engineered cell culture models.

## Introduction

Heart disease and cancer remain the top two causes of death in the US. One fundamental characteristic of both diseases is *tissue failure*: namely, errors in the structural organization and function of cells in the affected tissues. Traditional approaches for uncovering the source of these errors have relied heavily on reductionist approaches (e.g., genomics, proteomics, gene expression microarrays), yielding tremendous amounts of information about the genetic and biochemical makeup of these cells. Yet, the fundamental question remains: exactly what cellular structures and functions initiate the transition from healthy to diseased tissues, and why? We believe that one reason we have yet to answer this question is that the structure/function paradigm has developed a “gap” at this critical cell-to-tissue level; we have accumulated more information than we can integrate into a cell/tissue-level understanding of disease, such that the abundance of genetic and biochemical details are not being fully utilized to uncover how cells and tissues function. Likewise, we have an abundance of markers for many diseases, but we don't fully understand the rules that link the molecular constituents of diseased tissues to the clinical symptoms of the disease itself.

A dramatic example of this problem lies in the detection and diagnosis of cancer, where, despite a multitude of genetic screens, biochemical assays, and imaging techniques, the “gold standard” for diagnosis remains the expert opinion of highly trained pathologists who visually scan samples of the tissues in histology slides. In other words, the *human eye* is currently the most accurate tool we have available for identifying telltale alterations in the structure and function of diseased tissues. The same is true for diagnosis of heart disease, arthritis, and most other debilitating diseases. We believe that a more rigorous approach to linking the structural organization of healthy and diseased tissues to fundamental cellular behaviors will help close the gap in tissue structure/function, and provide clinicians a powerful tool for more accurately detecting and diagnosing disease.

Numerous techniques have been developed to extract information at the molecular, cellular, tissue or organ level to distinguish and classify distinct disease types, such as tumor types in cancer but none of these approaches can model the structure-function relationship in tissues as we discuss in the next section. In this paper, through the use of graph theoretical tissue representation [1], we model the structure-function relationship in tissues. Furthermore, our approach combines this representation with matrix and tensor factorization methods in order to identify sets of structural properties that discriminate between healthy and cancerous forms of three morphologically distinct tissues (brain, breast, and bone) and quantify the structural differences between these tissues and their tissue engineered counterparts. Both histological images of human tissue samples and fluorescence images of three-dimensional cultures of human cells are used to compare the accuracy of in vitro culture models (3D cell cultures and in vitro samples will be used interchangeably in the manuscript) with their corresponding human tissues (referred to as histology samples throughout the paper). To the best of our knowledge, there is no prior work on a quantitative comparison of histology and in vitro samples.

## Methods

### 1. Tissue and Cellular Analysis

In the literature, four different types of approaches have been used to define quantitative features at the cellular and tissue levels. The first uses morphology to quantify the size and shape of a cell or its nucleus [2]–[7]. The second employs intensity or the distribution of the color values of pixels to define features [8]–[11]. The third exploits textural descriptors and considers spatial dependency of the intensity values to quantify the smoothness, regularity or coarseness of the image [2], [5], [6], [12]–[17]. Finally, the fourth approach, which most closely resembles ours, is based on drawing a Voronoi graph of cells from a tissue image and computing graph-theoretical features that quantify how the cells are distributed over the tissue [7], [8], [18]. Nevertheless, none of these approaches can model the structure-function relationship in tissues.

### 2. Cell-graph Mining of Tissue Structures

Recently we introduced a powerful technique called the cell-graphs to model structural organization of histology tissue samples. Cell-graphs capture the characteristic structural properties that distinguish healthy, damaged, and cancerous states of brain [1], [19], [20], [21], breast [22], and bone tissues [23]; and properly classify follicular lymphoma [24]. We further extended this method for in vitro studies to model mesenchymal stem cells in three dimensional space [25], to ECM interactions during cell-mediated compaction and collagen remodeling in 3D [26], and to in vitro cancer data analysis, as explained in [27]. We also showed preliminary results of the applicability of cell-graph technique for capturing the distinctive epithelial and mesenchymal features in an embryonic branching organ – the salivary gland [28].

#### 2.1 Data Acquisition

In this manuscript we used the histopathology data as explained in our previous publications [1], [19]–[22] and in vitro cancer data [27]. Below we summarize the basic techniques and details of the data.

• Histopathology Data Acquisition: Our data has two different functional states (healthy and cancerous) of three different tissue types: brain, bone and breast. The data used in the study follows the specifics below:

Our **brain tissue** data set is a mixture of healthy tissue and diseased (glioma) samples. For preliminary studies, these tissues are randomly selected by a neuropathologist from Oregon Health and Science University (OHSU) Pathology Department archives, arbitrarily limiting the search to the years 2001–2004, and selecting well-preserved, technically adequate samples that best represent the different tissue states mentioned earlier, without excluding any particular patient population. Healthy tissue samples were taken when available from surgical specimens or autopsies. All diseased (glioma) samples were high-grade; and glioblastoma, anaplastic astrocytoma and anaplastic oligodendoglioma were included as diagnostic categories. Each sample consists of a 5–6 µm thick tissue section stained with hematoxylin and eosin (H&E) technique and mounted on a glass slide. In each case, a representative H&E-stained glass slide was chosen by the pathologist, and the patient identifiers (i.e. the accession numbers on slide labels) were removed after diagnostic tabulation in a coded manner. Subsequently, digital photomicrographs of different fields of the lesion were obtained in a standardized way in a Nikon Coolscope digital camera/scanner by the pathologist. Uniformity of the images was obtained by keeping the magnification and illumination at selected levels, allowing sufficient resolution to detect individual tumor cell nuclei. Special attention was given to avoid areas of hemorrhage and necrosis, treatment effects, blood vessels and tissue artifacts. Different sets of pictures were obtained of the tumor parenchyma, interface of the tumor and the surrounding brain, and histologically normal areas when available. Areas of low and high cellularity were also sampled as images in different entities. Prior to segmentation, we converted the RGB values of pixels to their corresponding values in the La*b* color space. Unlike the RGB color space, the La*b* color space is a uniform color space and the color and detail information are completely separate entities. Therefore, using the La*b* color space yielded much better quantization results. Data set in this study contains 210 images of 14 patients with healthy histology samples and 329 images of 41 patients with malignant glioma histology samples.

The data set for **breast tissue** modeling was randomly selected from the archived Mount Sinai School of Medicine (MSSM) Pathology Department archives. The two different states of breast tissue cases were reviewed by two breast pathologists at MSSN to reach a consensus. The data set, we used in this study contains 128 invasive cancerous tissue images of 19 patients, and 195 healthy tissue images from 19 patients.

The data for **bone tissue** modeling and classification was provided by the Pathology Department at Hospital for Special Surgery (HSS) in NYC. H&E stained images of two different states of bone tissue (healthy and diseased [cancerous]) were collected with 10× magnification. The data set used in this study contains 20 images of healthy bone tissue, and 49 images of osteosarcoma (diseased) bone tissue.

• In Vitro Data Acquisition: Regarding to the cell culture techniques, the different cell types and their respective culture conditions are listed in Table 1 and the functional categories of each cell type are listed in Table 2 of our previous work in [27]. For fluorescence imaging, gels were fixed using 3% paraformaldehyde at 11 different time points (hours): 0, 1, 2, 4, 6, 10, 16, 24, 72, 120, 168. Each was washed with PBS, then stained with nucleic acid dye (sytox green). Images of cells encapsulated within collagen-I hydrogels were captured using a Zeiss LSM 510 META confocal microscope with a 10× dry objective. Representative Z-stack images of 100 µm thickness with 900 µm×900 µm cross-section area were collected for five samples of each time point.

#### 2.2 Cell Graph Construction and Feature Extraction

**Figure 1:**
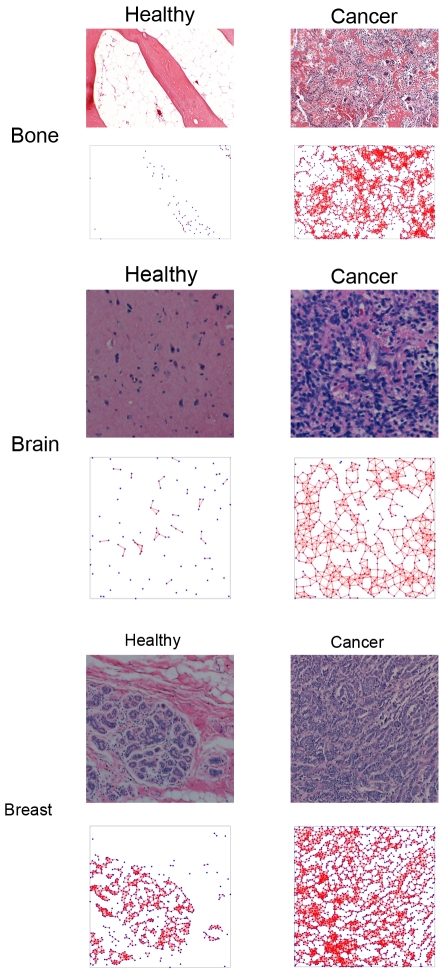
Examples of different tissue types and states as well as their representations as cell-graphs.

Our overall methodology used for obtaining the cell-graphs in this work can be summarized in two phases. First, we build 2D cell-graphs to represent a tissue state (Figure 1 illustrates the cell graphs for different tissue types and states). Second, the graph theoretical features of these cell-graphs are computed.

*In cell-graph generation phase*, we have three steps: (i) color quantization, (ii) node identification, and (iii) edge establishment. Details of these steps can be found in our cited publications above. We explain these steps briefly here:

• Image Processing and Segmentation: In this step we used standard image processing tools to distinguish the cells from their background based on the color information of the pixels. For that we use the k-means algorithm. The k-means algorithm clusters the data based on their features. There are k cluster vectors and each sample is assigned to its closest cluster and represented with this clustering vector. Subsequently, each of these clustering vectors is assigned either to be “cell” or “background” class by the pathologists.

• Node Identification Step: In a cell-graph, cells or cell clusters of a sample tissue are the vertices. We have several methods to extract vertices of a cell graph from data. The method applied to the histology data used in this manuscript can be described as follows: In the node identification step, we translate the class information of the pixels to the node information of a cell-graph. In this step, we have two control parameters: (i) the size of the grid, and (ii) the threshold value. The grid size determines the down sampling rate, i.e., the resolution of the resultant image. After this step, a node can represent a single cell, a part of a cell, or a bunch of cells, depending on the grid size. We embed a grid over the tissue image and assign a value of 1 to the pixels of “cell” class and a value of 0 to the pixels of “background” class. Subsequently, for each grid entry, we compute a probability of being a cell or background by computing the average values of pixels located in this grid entry. At the end of this step, the spatial information of the cells is translated to their locations in the two-dimensional grid. After computing the probabilities, we compare these against a threshold value. The grid entries with a probability value greater than the threshold are considered as the nodes of the cell-graph. Thus, the second control parameter is the threshold value, which eliminates the noise that arises from the stain artifacts and mis-assignment of black pixels in the color quantization step.

Considering the large number of images that need to be processed, we employ Otsu's simple but effective automatic threshold selection algorithm that determines a global (single) threshold for the image based on the histogram of image values. Each connected component in the resulting binary image corresponds to a nucleus and the coordinates of the centroids of these nuclei are calculated to identify the coordinates of the node (vertex) set for cell-graph generation.

• Edge Establishment Step: In the edge establishment step, we set the links between the nodes to generate a cell-graph. Formally, let 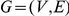 denote a cell-graph with V and E being the set of nodes and edges of the graph, respectively. After determining V in the node identification step, we define an edge (u, v) between a pair of nodes u and v by making use of the biological insight and knowledge on the interaction of the cells in a specific tissue type. For example, it may be more likely that physically adjacent cells signal each other than the ones far away. Such distance based interaction among the elements is well understood in physical systems based on energy minimization. In the absence of multiple markers (recall that images are H&E stained) we rely on a proximity based establishment of pairwise relationships between nodes. Therefore, we translate the pairwise spatial relation between every two nodes to the possible existence of links in a cell-graph. We can establish the edges probabilistically or deterministically or use a combination of these two methods. For example, in [1] we constructed probabilistic cell-graphs in which the probability of creating a link between any two nodes decays exponentially with the Euclidean distance between them with a function 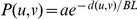, or with a power law probability function such that 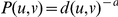 where d(u, v) is the distance between these nodes. Intuitively, the closer two cells are, the more likely that they share a relationship. This probability quantifies the possibility for one of these nodes to be grown from the other thus, aiming to model the prevalence of the disease state in a tissue.

An edge (u, v) can also be deterministically established if the distance d(u, v) is less than a threshold (e.g., two cells are physically touching each other). This is indeed the method used for constructing the cell-graphs analyzed in this paper. The motivation is that if cell membranes are touching or close enough (we quantify this by parametric search) then there is some signaling between them. We have identified link thresholds, corresponding to the approximate radii of spread mammalian cells, of 65, 70, and 75 microns (from a distortion free sphere representation of the cell membrane to observable distortion) and performed parametric search to maximize the classification accuracy of our modeling. Note that the presence of a link between nodes does not specify what kind of relationship exists between the nodes (cells); it simply indicates that a relationship of some sort is proposed to exist, and that it is dependent on the distance between cells. Surprisingly, the distance measure alone is sufficient to reveal important, diagnostic structural differences in human tissues.

If the images carry multichannel information by applying more sophisticated staining techniques (e.g., multispectral fluorescence imaging), it is possible to build cell-graphs that have different types of nodes corresponding to different types of cells that co-exist (e.g., epithelial vs. fibroblast) and other ECM entities (e.g., basement membrane underlying epithelial cell layers and blood vessels). With 3D images and 3D cell-graphs, such representation becomes more accurate and powerful; this is indeed currently investigated by our group.

The second phase is *feature extraction* from cell graphs. The cell-graphs enable us to apply well-established principles of graph theory and provide a rich set of features defined precisely by these principles to be used as quantitative descriptor features. They can be classified into three groups: *connectivity* indicators such as degree, clustering coefficient, giant connected component; *distance* indicators such as diameter, radius, hop plot exponent; *compactness* indicators such as closeness, central points, isolated points, link lengths. We showed in our previous work that these features obtain different values based on different functional states thus one can train classifiers to quantify the relationship between feature values and functional states [1], [19]–[27]. The features we included in this paper are the ones used commonly in our previous work and listed in Table S1.

We consider two types of features: (i) local features at the individual cell level, and (ii) global features at the tissue level to be used by the algorithms to distinguish different tissue types. By computing the distribution of local features, we can obtain global features. However, some global features can only be computed over the entire graph. For example the ratio of the size of the giant connected component over the size of the entire graph can be used as a global feature. Other global features are related to the spectrum of a graph, which is the set of graph eigenvalues computed from the adjacency matrix or its Laplacian. The spectral radius and eigen exponent are such features. The eigenvalues of the Laplacian relate to the graph invariants better than the eigenvalues of the adjacency matrix. For example, the number of eigenvalues with a value of 0 gives the number of connected components in the graph. Moreover, as the eigenvalues of the Laplacian lie in the range of [0,2], it is easier to compare the spectra of graphs with different sizes. We also use global features to characterize the spectra of cell-graphs, i.e., the eigenvalue distribution of the Laplacian of the cell-graph. While some of these features are easier to relate to underlying biology such as degree, closeness; some others are hard to associate with biology such as the spectral one. However, these features collectively describe the structural organization of underlying tissue sample as we demonstrated in our previous work.

### 3. Matrix and Tensor Factorizations

**Figure 2:**
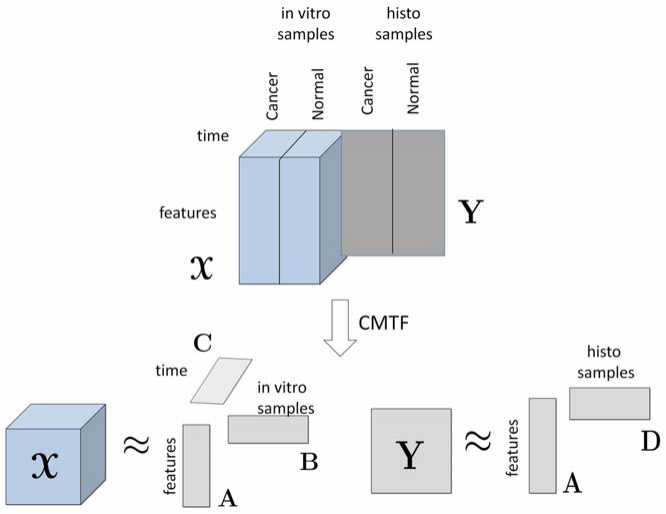
Analysis of histology and in vitro data sets using coupled matrix and tensor factorization (CMTF). Time mode is slotted as 0, 1, 2, 4, 6, 10, 16, 24, 72, 120, 168 in hours. Features mode contains the cell graph features: average degree, clustering coefficient C, clustering coefficient D, clustering coefficient E, average eccentricity, diameter, radius, average eccentricity 90, diameter 90, radius 90, average path length, effective hop diameter, hop plot exponent, giant connected component ratio, # connected components, average connected component size, % isolated points, % end points, # central points, % central points, mean, std, skewness, kurtosis, # nodes, # edges. These features are defined in Table S1.

Using cell-graph features, the data can be arranged as a matrix (cell-graph features by histology samples) or a three-way tensor (cell-graph features by in vitro samples by time) (see Figure 2). We analyze the data arranged as in Figure 2 to (i) differentiate between different tissue functional states, (ii) identify the features responsible for such differentiation, and (iii) discriminate between histology samples and their corresponding in vitro engineered cell cultures using matrix and tensor factorizations.

**Figure 3:**
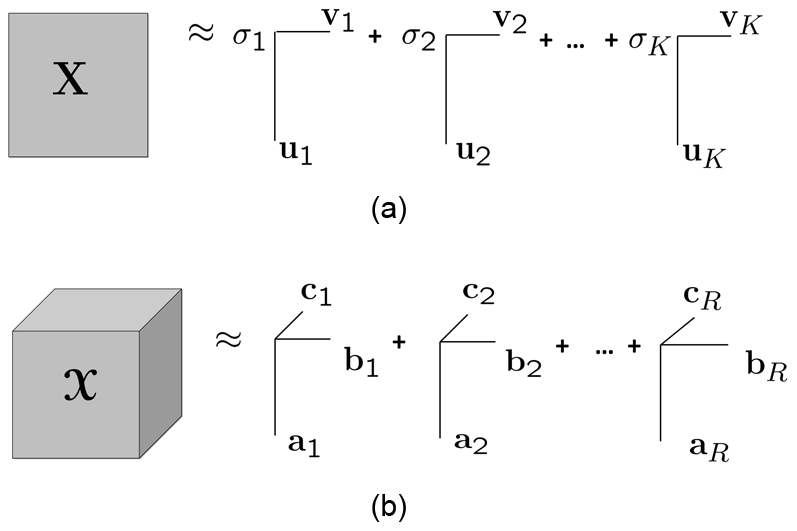
Singular Value Decomposition and R-component CP model.

Matrix factorizations, in particular Singular Value Decomposition (SVD) [29], are commonly used for exploratory data analysis to extract the underlying factors in complex data sets [30]. Given a matrix 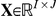 of rank *R*, SVD computes orthogonal matrices 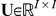 and 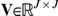 such that 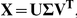 where 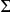 is a diagonal matrix with *σ*_1_, *σ*_2_, *…σ_R_* on the diagonal and *σ*_1_≥*σ*_2_≥*…*≥*σ_R_*. The columns of 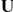 and 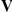 are the *left* and *right singular vectors*, respectively, and the diagonal entries of 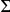 are the *singular values*. In order to find a low-rank approximation of the data, we can use only the first *K* (*K<R*) singular values and vectors; and this gives the best rank-*K* approximation of the data (Figure 3a).

Tensor factorizations are generalizations of matrix factorizations to higher-order tensors. An *N*-way tensor (or an *N*th-order tensor) is a multidimensional array represented using *N* indices, e.g., a vector is a first-order tensor; a matrix is a second-order tensor. *N*-way arrays, for 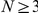, are called higher-order tensors. Here, we use one of the most popular tensor models, i.e., CANDECOMP/PARAFAC (CP) [31], [32], which has proved useful for finding the underlying structures of higher-order data sets in various disciplines such as chemometrics, computational neuroscience and social network analysis [33], [34]. Given a tensor 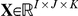, its *R*-component CP factorization is expressed as follows:
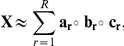
where 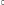 denotes the vector outer product, and 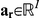, 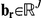, and 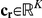 for *r = 1,…,R*. The matrices 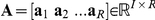, 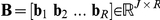 and 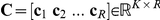 correspond to the CP *factor matrices (component matrices)* extracted from the first, second and third *mode* (or *dimension*) of the tensor, respectively. We use the compact notation, 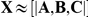 to denote the CP model [34]. Just as SVD represents a matrix as a sum of rank-one matrices, the CP model expresses a tensor as a sum of rank-one tensors (Figure 3b).

### 4. Joint Analysis of In vitro and Histology Samples

While clinical biopsies (histology samples) represent the current standard for determining human tissue state, their limited availability, sample variability, and high cost are often prohibitive for studying the underlying mechanisms that control tissue function. Despite their limited simplicity, three-dimensional engineered cultures of human cells grown in vitro offer the advantages of providing complete control over environmental conditions and permitting invasive analyses that are difficult or impossible to perform with human subjects. The costs and benefits of using histology and in vitro samples are therefore an important consideration in any study of tissue structure and function. In this study, we used image data from both sources.

Joint analysis of data from multiple sources can improve our understanding of the underlying structures in complex data sets. For instance, we represent our in vitro samples as a set of features changing over time, which forms a third-order tensor with modes: *features* by *samples* by *time*. We express our histology samples using the same set of features as a *features* by *samples* matrix. By analyzing these two datasets (arranged as in Figure 2) jointly and extracting the same factors from the features mode, we may capture the common dynamics in both in vitro and histology samples. We can then use these dynamics to differentiate between different tissue functional states or to understand what features are influential for differentiating between those functional states.

We analyze the two data sets in Figure 2 using coupled matrix and tensor factorizations [35]. Given a third-order tensor 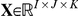 and a matrix 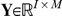
_,_ the tensor and the matrix are factorized jointly using coupled matrix and tensor factorization (CMTF), where an *R*-component CMTF model of a tensor 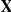 and a matrix 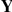 can be computed by solving the following optimization problem:
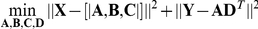
where 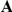 is the factor matrix corresponding to the common mode, i.e., features modes; 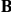, 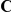,and 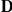 correspond to factor matrices in other modes. 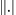 denotes the norm of a tensor and it is defined as 
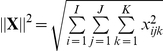
. We solve the optimization problem by solving the problem for all factor matrices simultaneously, i.e., the gradient is computed by taking the partial derivative of the objective function with respect to each factor matrix and concatenating the derivatives and then we use Nonlinear Conjugate Gradient to solve the CMTF problem [35].

## Results

### 1. Normal vs. Cancer: Brain Tissues

Our first objective was to test whether the same cell-graph feature set we used to classify biopsy (histology) samples of brain [1], breast [22], and bone [23] tissues would be sufficient to permit us to properly segregate 3D engineered cell cultures containing healthy or cancerous cells from the same tissues [27]. These cultures are simpler than their native counterparts, because they contain only one cell type, and thus cannot recapitulate the full structure or function of their tissue of origin. However, because they begin as diffuse clusters of cells encapsulated in a collagen gel, they are far more disorganized than even the most cancerous tissues, and thus undergo a complex series of morphological changes (e.g., gel compaction, establishment of cell-cell junctions, etc.) that do not normally occur in histology samples. In fact, most modern 3D cell cultures undergo transformations that more closely resemble wound healing than embryonic development.

**Figure 4:**
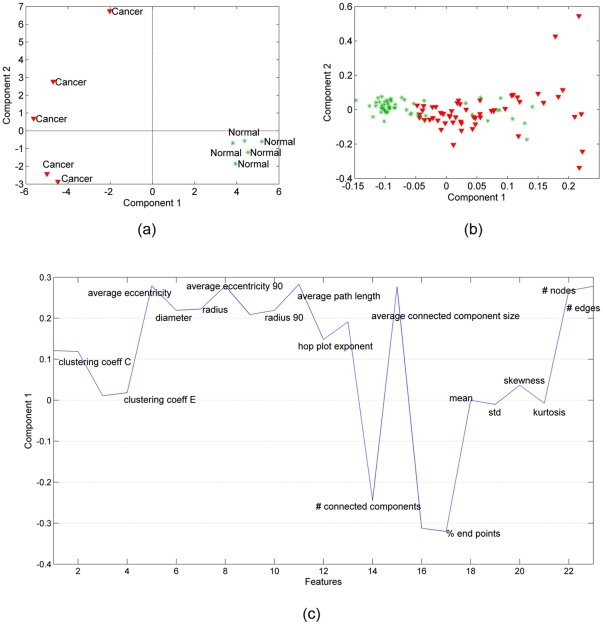
Three-way and Two-way analysis of in vitro brain tissue data. (a) CP factorization of the tensor with modes: *features, samples* and *time*. The 1st component separates the 2 different functional states: cancer (red-triangle sign) from normal (green-plus sign) tissue samples; (b) SVD of matrix of type: *features* by *samples (across all times)*; (c) features projected over the 1st component of the CP model. Cell-graph features such as *% of end points, number of connected components, average connected component size, average path length, average eccentricity* are identified as influential in the analysis since their coefficients diverge the most from zero. (Note that we have 23 features on the plot since three features have been identified as outliers and excluded).

Using cell-graph features, we arrange in vitro tissue samples measured at different time points as a *features* by *samples* by *time* tensor. Let 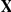 denote this tensor. We compute its CP factorization, i.e., 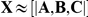, and extract the factor matrices **A**, **B** and **C** corresponding to the features, samples and time modes, respectively. In our analysis, a 2-component CP model is used and the number of components is chosen based on the core consistency diagnostic [36]. We focus on the component matrices in features and samples mode. Figure 4a shows the scatter plots of the factors in the samples mode, i.e., 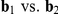. Figure 4c illustrates the first factor in the features mode, i.e., 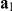, since the first component differentiates between cancerous and healthy samples in Figure 4a. We also matricize tensor 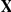, in the first mode by arranging the tensor as a *features* by *samples-time* matrix (see [33] for matricization), and compute its SVD. Figure 4b shows the scatter plot of the right singular vectors, i.e.,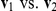.

The results in Figure 4 illustrate three important aspects of our analysis. First, healthy and cancerous brain cells grown in 3D culture (in vitro samples) can be discriminated when we represent them using cell-graph features and take into account how they change in time. Three-way analysis can easily discriminate between them (Figure 4a) (We also illustrate that it is not possible to separate brain cells at every time sample as healthy or cancerous (Figure 4b)). Second, this discriminative power relies primarily on a small subset of the graph features, suggesting they may contain the telltale signatures of functional state, even in 3D monoculture. These features agree well with our understanding of the cellular and molecular changes that occur during malignant transformation. For example, the features *# connected components*, *% end points*, and *average connected component size* reflect the degree of “communication” between nodes in a cell graph; as healthy brain becomes cancerous in vivo, much of this communication is lost due to loss of synaptic junctions, although the relative position of the cell bodies may not change much. As the tumor cells proliferate, the increased number of cells becomes an obvious distinguishing feature, reflected in our analysis by the metric *# nodes*. By comparison, the *mean* indicating the average edge length is of relatively low diagnostic value, and contributes very little to the first component of our three way analysis. Third, because these features are also quantitative, we now have a statistically rigorous means of classifying functional state based on the same raw data that generate qualitative classifications in the clinic.

**Figure 5:**
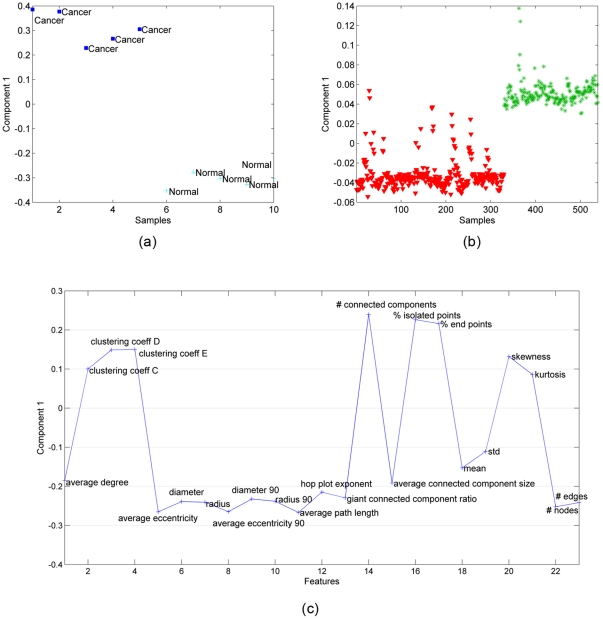
Coupled matrix and tensor factorization (CMTF) on in vitro brain samples represented by tensor 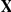
 **and histology samples represented by matrix Y (see** **Figure 2** **).** (a) The first column of matrix **B**, that is the factor matrix corresponding to the in vitro samples mode extracted using CMTF, separates cancer (blue-square signs) from normal (light blue-plus signs) tissue samples; (b) The first column of matrix **D**, that is the factor matrix corresponding to the histology samples mode extracted using CMTF, can separate cancer (red-triangle sign) from healthy (green-star sign) samples; (c) features captured by the common component extracted by CMTF.

We gain additional insight by adding the histology samples to our analysis. We represent them as a matrix of type: *features* by *samples*, using the cell-graph features. This yields two sources of information: (i) in vitro samples arranged as a tensor (see tensor 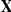 in Figure 2) and (ii) histology samples arranged as a matrix (see matrix **Y** in Figure 2). These data sets are coupled in the features mode in that they use the same set of cell-graph features. Using coupled matrix and tensor factorizations, we factorize tensor 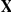 and matrix **Y** in such a way that 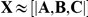 and 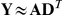, where **A** corresponds to the common factor matrix in the features mode. **B** and **C** represent the factor matrices for samples (in vitro) and time modes, respectively while **D** is the factor matrix corresponding to the histology samples. In coupled analysis, we observe that there is only one common component (Extracting more components results in degenerate models, where one component is highly negatively correlated with another component - see [34] for a discussion on degeneracy in the case of tensor factorizations). Figure 5a plots the factor vector in the in vitro samples mode, i.e., 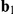, while Figure 5b plots the factor vector in the histology samples mode, i.e., 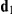. The common factor can differentiate between cancer and healthy samples in both Figure 5a and Figure 5b. In Figure 5c, we illustrate the common factor in the features mode, i.e., 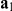, in order to see the features responsible for the differentiation of functional states.

The results in Figure 5c further demonstrate that subsets of graph features can successfully separate distinct functional states in structurally similar tissues. Note that three-way analysis and coupled analysis produce similar feature sets to distinguish healthy and cancerous forms of human tissue samples. This is expected since our analysis targets only the spatial distribution of nuclei (although tissue biopsies contain far more structural complexity than 3D in vitro monocultures of healthy and cancerous cells,). The *# connected components*, *average connected component size*, and *giant connected component ratio* are global features that capture patterns across the entire graph.

### 2. Normal vs. Cancer: Bone Tissues

**Figure 6:**
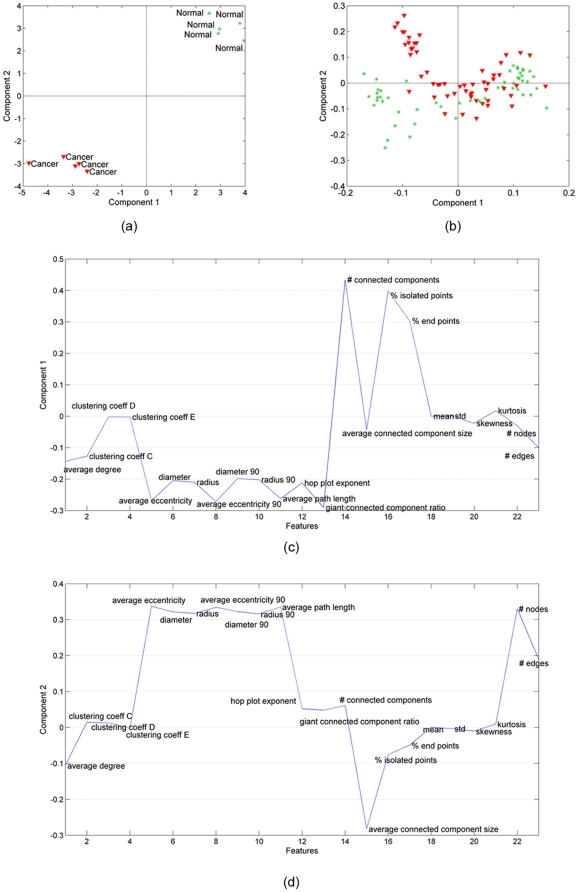
Three-way and two-way analysis of in vitro bone tissue data. (a) CP factorization of the tensor with modes: *features*, *samples* and *time*. Both the 1st and the 2nd components separate the two different functional states: cancer (red-triangle sign) from normal (green-plus sign) tissue samples; (b) SVD of matrix of type: *features* by *samples (across all times)*; (c) features projected over the 1st component of CP model. Cell-graph features such as *% of end points*, *number of connected components*, *giant connected component ratio, average path length, average eccentricity* are identified as influential in the analysis since their coefficients diverge the most from zero; (d) since the 2nd component can also distinguish between two functional states we also show the 2nd CP component in features mode. Note that the influential features are different in the 2nd component, e.g., while the *number of connected components* has a high coefficient in the 1st component, its coefficient in the 2nd component is close to 0.

We repeated the analysis for bone samples (obtained in our previous study [23]) by constructing a third-order tensor with modes: features, samples, and time, and computing its CP factorization. Similar to Figure 4a, in Figure 6a, we show the scatter plots of the factors in the samples mode. We observe that both the first and second factors can separate the healthy and cancerous in vitro samples. Figure 6c and 6d, therefore, illustrate the first and second factors in the features mode. As in Figure 4b, Figure 6b shows the scatter plot of right singular vectors of tensor 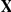 matricized in features mode. The results in Figure 6 are similar to those in Figure 4, in that three-way analysis can separate the healthy and cancerous in vitro samples. This is significant because it demonstrates that the discriminative power of our analysis is independent of cell/tissue type. While brain and bone tissues are morphologically quite distinct, our analysis reveals they share many of the same core structural features. When we compare the significant features in Figure 6c to those in Figure 4c, we see a great deal of overlap, suggesting that the first component of our CP analysis of bone cultures exhibits a discriminative power similar to the first CP component for our in vitro brain samples. In addition, the second component of our in vitro bone analysis (Figure 6d) contains additional significant features.

**Figure 7:**
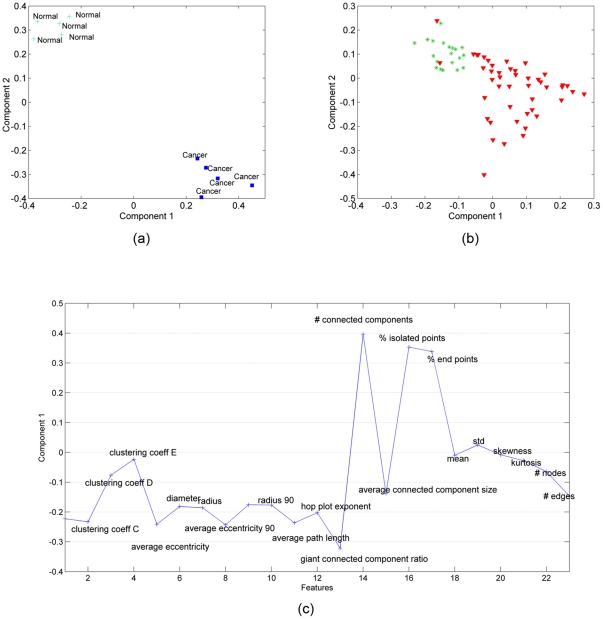
Coupled factorization of in vitro bone samples represented by tensor 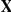
 **and histology samples represented by matrix Y.** (a) Both the 1st and the 2nd column of matrix **B** extracted by a CMTF model separate cancer (blue-square sign) from normal (light blue-plus sign) samples; (b) Matrix **D** corresponding to the histology samples mode extracted using a CMTF model is useful to narrow the coupled analysis since only the 1st component can separate cancer (red-triangle sign) from healthy (green-star sign) samples; (c) features captured by the 1st CMTF component.

In Figure 7, we illustrate the results of coupled analysis of in vitro and histology samples. In this case, it is possible to extract two common factors. Figure 7a plots the factors in the in vitro samples mode, i.e., 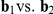, while Figure 7b plots the factor vector in the histology samples mode, i.e., 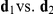. The first common factor can differentiate between cancer and healthy samples in both Figure 7a and Figure 7b; therefore, we illustrate 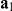 in Figure 7c to show the features responsible for the differentiation in both histology and in vitro samples. A comparison of Figure 5 and Figure 7 illustrates that our coupled matrix-tensor analysis is equally powerful for classifying in vitro brain and bone samples, and that this classification relies on a similar set of features for both tissue types. This strongly suggests that our analysis of combinations of cell-graph features from histology and in vitro samples, even from morphologically distinct tissues, converges on a core set of features that serve as *tissue signatures*, defined in large part by the global organization of the entire tissue. Note that while the absolute values of these features may differ considerably between different tissue types, we feel the fact that these signatures are composed of a small set of features uncovers a key underlying organizational principle in tissue structure and function, and thus may be important for closing the gap in our understanding of tissue structure and function.

### 3. Normal vs. Cancer: Breast Tissues

**Figure 8:**
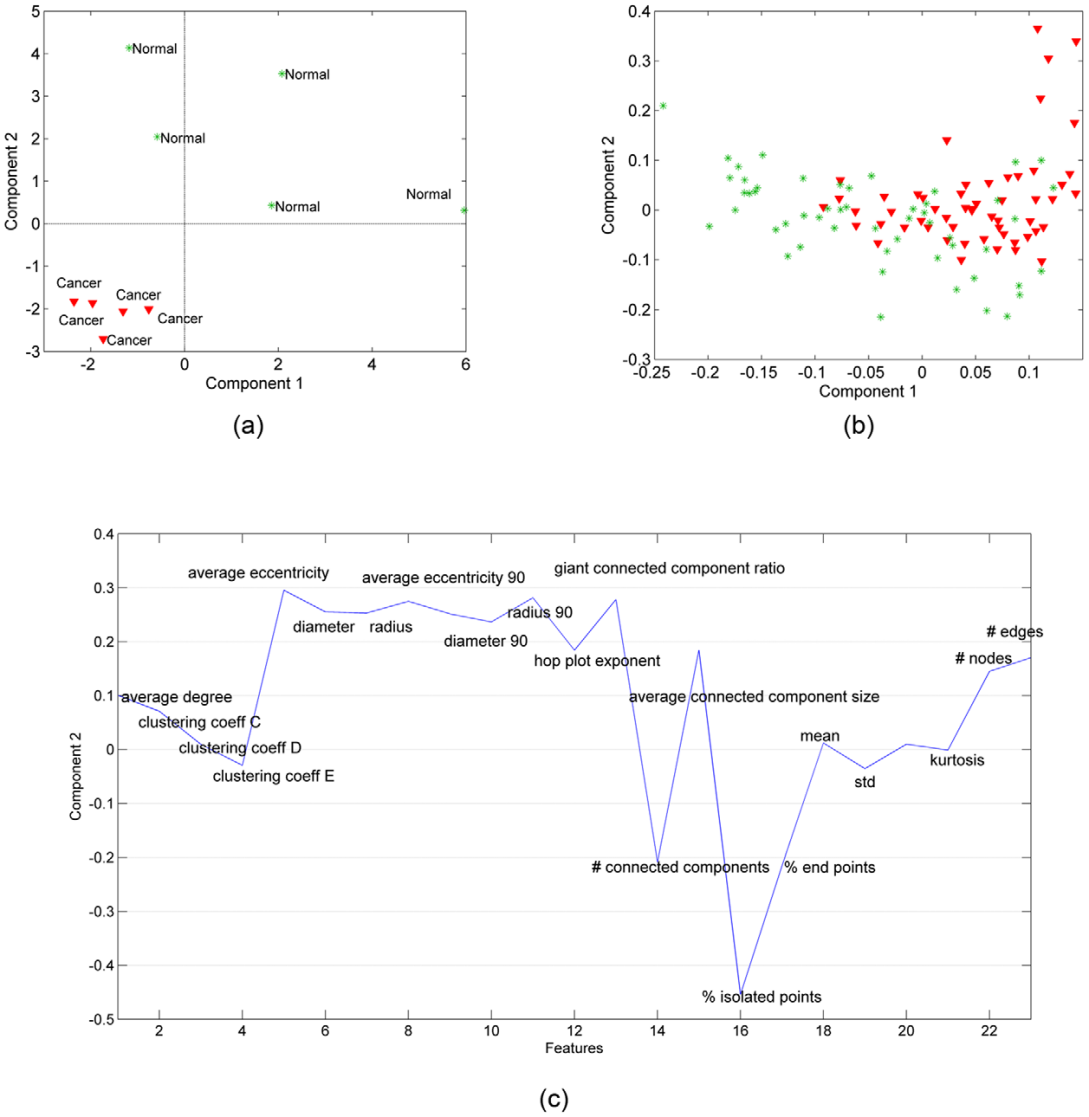
Three-way and two-way analysis of in vitro breast tissue data. (a) CP factorization of the tensor with modes: *features*, *samples* and *time*. Only the 2nd component can separate the two different functional states: cancer (red-triangle sign) from normal (green-plus sign) tissue samples; (b) SVD of matrix of type: *features* by *samples (across all times)*; (c) features projected over the 2nd CP component. Cell-graph features such as *% of end points, number of connected components, average connected component size, average path length, average eccentricity* are identified as influential in the analysis.

Figure 8 extends our conclusions from Figures 4 and 6 to include a ductal tissue as well. Of the three tissue types we examined, breast tissue structure is by far the easiest to understand intuitively: it is primarily organized into ducts lined with secretory cells, and clear boundaries (the basement membranes) separate these secretory cells from the remainder of the cells in this tissue. Our 3D monocultures of normal breast cells recreate this epithelial architecture quite well, including the basement membranes. In contrast, our breast cancer cell cultures are far more diffuse, reflecting the loss of structural integrity in breast tumors. It is perhaps somewhat surprising, therefore, to see that our three-way analysis is necessary to discriminate between the two sets of in vitro samples, and that only the second component of this analysis could achieve true separation. This is important, because it indicates that our graph features are not simple abstractions of patterns immediately visible to trained pathologists. Instead, we feel these features reflect an underlying organizational theme that all tissues follow, regardless of their visible appearance.

**Figure 9:**
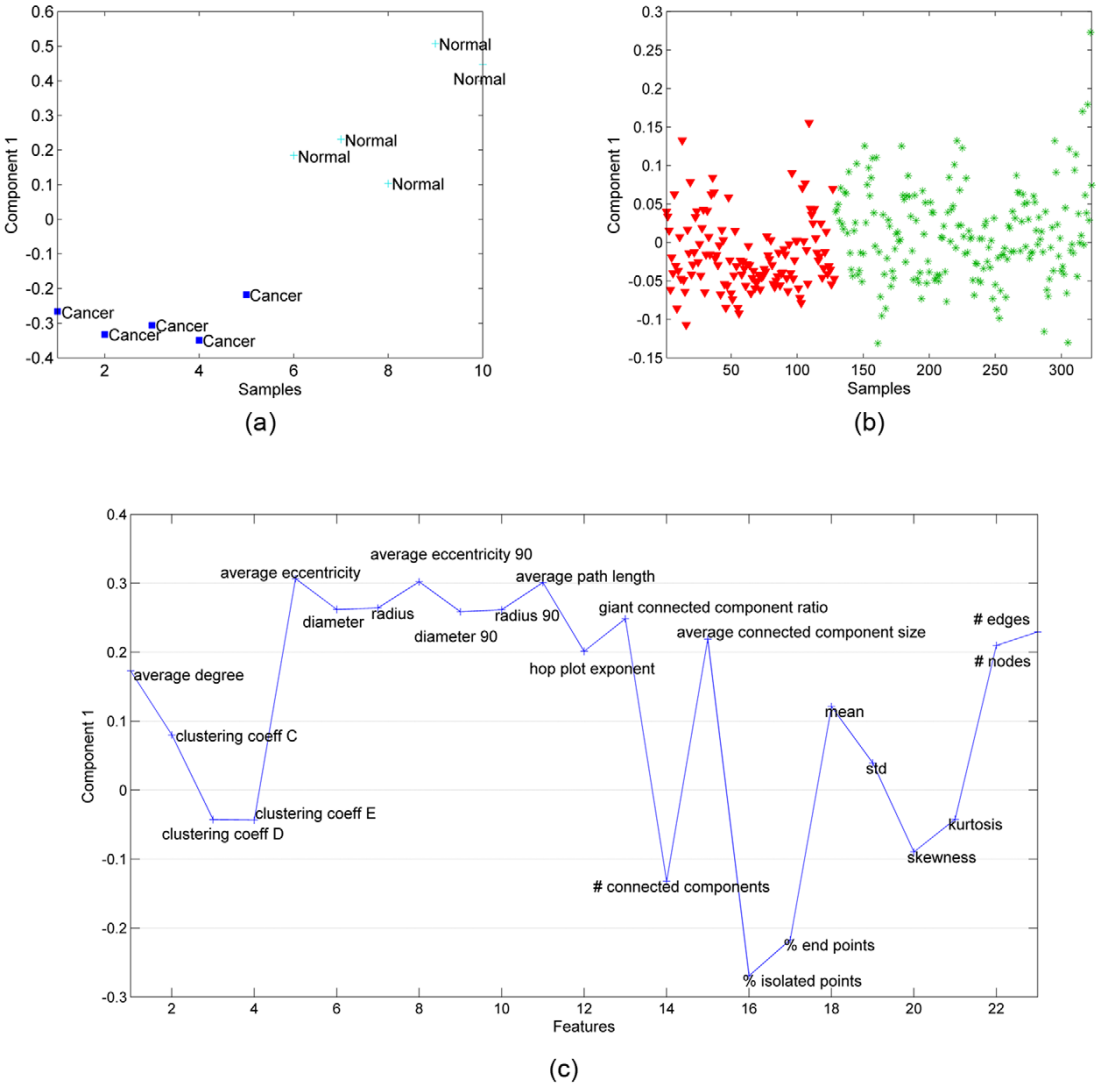
Coupled matrix and tensor factorization on in vitro breast samples represented by tensor 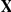
 **and histology samples represented by matrix Y (** **Figure 2** **).** (a) The 1st column of matrix **B** corresponding to the in vitro samples mode extracted by a CMTF model can separate cancer (blue-square sign) from normal (light blue-plus sign) tissue samples; (b) Unlike for brain and bone tissues, matrix **D** corresponding to the histology samples mode extracted using a CMTF model cannot separate cancer samples (red-triangle sign) from healthy (green-star sign) samples; (c) features captured by the common component extracted by CMTF. Cell-graph features identified as influential in the coupled analysis are similar to the features in Figure 5c and 7c with some minor differences.

Figure 9 further underscores this point, in that we cannot discriminate between healthy and cancerous histology samples (Figure 9b), even when they are coupled to their corresponding in vitro samples; if the cell-graph features reflected obvious visible patterns, we would expect to segregate them. On the other hand, we find that the common component extracted by our coupled analysis (only one component is extracted using CMTF) is sufficient to differentiate between healthy and cancerous in vitro samples (Figure 9a). The most influential features in the common component are nearly identical to those that discriminate brain and bone samples. Collectively, these findings suggest to us that this small set of features may constitute a global signature for most, if not all, human tissues.

### 4. Clustering of In Vitro vs. Histology Samples

**Figure 10:**
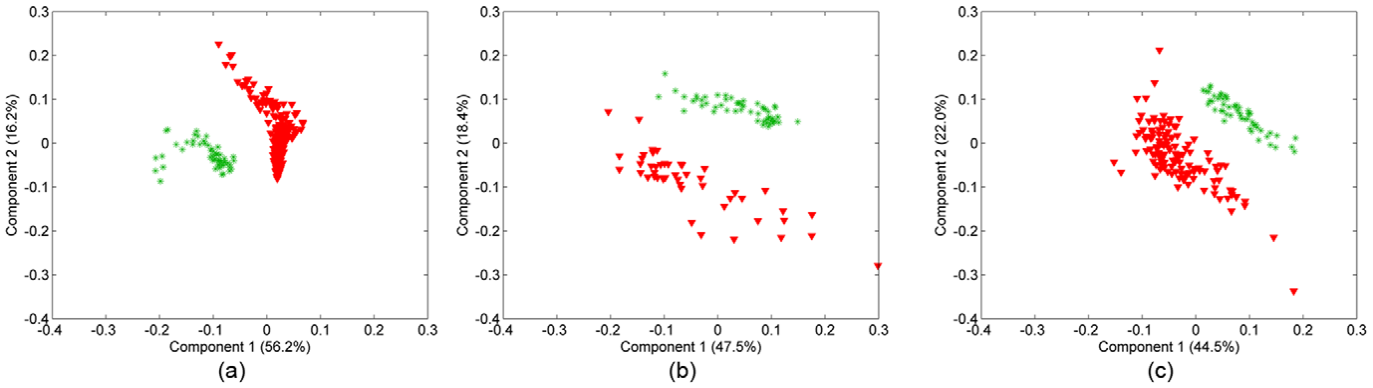
In vitro vs. histology samples of cancerous tissue (10a Brain, 10b Bone, and 10c Breast samples). The first two components of SVD analysis explain 72.4%, 65.9%, 66.5% of the variance for each tissue type, respectively. SVD yields a linear separation between in vitro and histology cancerous tissue samples. Two clusters (red and green) are very well separated, with few outliers. This defines and quantifies a structural difference between engineered tissues and the native tissues.

**Figure 11:**
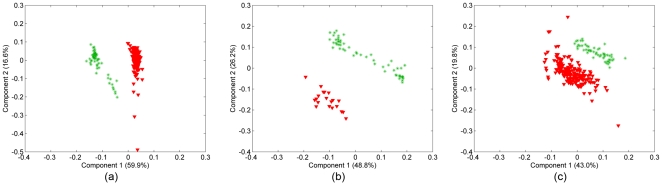
In vitro vs. histology samples of normal tissue (11a Brain, 11b Bone, and 11c Breast samples). The first two components of SVD analysis explain 76.5%, 75%, 62.8% of the variance for each tissue type respectively and shows that there is a linear separation of in vitro and native healthy tissue samples. The separation confirms that recreating the complex structural organization of native tissue samples in vitro is difficult.

Our second objective was to examine the organizational limitations of our 3D cell cultures vis-à- vis the actual tissues they represent. Let 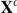 and 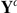 represent the cancerous samples in tensor 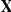 and matrix Y (see Figure 2). We matricize the tensor 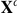 in the features mode as a features by samples-time matrix and then concatenate with 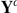 forming a new matrix 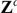 containing both cancerous in vitro and cancerous histology samples. The SVD of 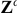 is computed to see whether we can extract factors that can differentiate between in vitro and histology samples. The first two right singular vectors of 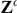 are plotted in Figure 10 for different tissue types. Figure 10 illustrates that all three of our in vitro cancer models are sufficiently different from actual tumors to permit SVD to clearly segregate them: the first two components of the SVD analysis capture 72.4%, 65.9%, and 66.5% of the explained variance for brain, bone and breast tissue samples respectively. The same is true for our healthy cell cultures (Figure 11): the first two components of SVD analysis explain 76.5%, 75%, 62.8% of the variance for each tissue type, respectively. Thus we conclude that in vitro sample and native histology samples contain quantifiably different structural organization of cells.

We examined the amount of cumulative explained variance by 3rd, 4th,.. 20th components to see if there is a significant difference between healthy and cancerous samples when we grow them in vitro vs. histology. We noticed that explained variance becomes almost equal for healthy brain and cancerous brain samples by using the first 5 components and thereafter. Similarly for breast tissue samples using the first 6 components and thereafter equal the explained cumulative variance for cancerous and health samples. In case of bone tissue the difference between in vitro and histology is more significant since cancerous tissue sample analysis requires always more components than healthy one for the same explained cumulative variance. This observation may indicate that organizing cells in vitro to obtain bone like tissue samples is a more challenging task.

### 5. Distance Between Time Evolving In Vitro Data and Histology Data

Based on our conclusions thus far, our next objective was to explore whether the accuracy distance between our 3D cultures and histology samples was time dependent. We hypothesized that because our 3D cultures undergo much more profound organizational changes than histology samples, their “accuracy” improves as they convert from an initial, evenly dispersed culture to a more mature structure, then become progressively less accurate as the cells undergo multiple rounds of mitosis and apoptosis (a product of the growth factors in the culture media and decreasing diffusion distance as the gels compact, respectively). This suggests that each culture reaches a “peak accuracy” over the course of a seven day incubation.

**Figure 12:**
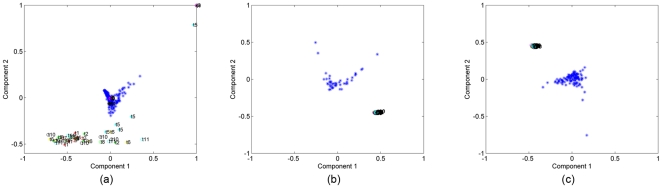
In vitro tissue samples remain structurally different from histology samples (blue) over time. We identified no time point in the development of the in vitro samples that is mathematically similar to histology data. (a) Brain cancer cultures vary considerably over time getting very close to the histology samples, demonstrating they best resemble the histology samples. In vitro bone cancer (b) and breast cancer (c) samples remain clustered over all time points and exhibit no mixing of data points with histology data.

To test this hypothesis, we compute the SVD of the cancerous histology samples and project the in vitro samples (at different time points) onto the first two singular vectors of the histology samples (after centering/scaling according to histology samples). Figure 12 shows that development of all three in vitro tissue types remains significantly different from histology samples, and quantifies this difference between in vitro and native tissue organization. In addition, while our bone and breast cultures (panels b and c) do not move closer to their histology sample clusters over time, the distance of brain cultures varies considerably with time getting very close to the histology samples, demonstrating they best resemble the histology samples. These data support our hypothesis for our brain cultures but fail for bone and breast.

## Discussion

There is a huge amount of data collected about biological tissues at the cellular and subcellular level. However, we still do not have an understanding of what these structural data corresponds to in terms of biological functions. In order to address this issue and improve our understanding of structure/function relationship, in this paper, we use a set of cell-graph features (see [37] for a review article) that are capable of modeling the structure/function relationship in tissues and construct matrices and third-order tensors representing histology and in vitro samples, respectively. Using tensor analysis of in vitro samples, for three different tissue types, i.e., brain, bone and breast, we have demonstrated that it is possible to discriminate between healthy and cancerous brain cells grown in 3D culture using a small set of graph features. Besides, we have shown that we can gain additional insight by incorporating histology samples and modeling matrices and third-order tensors jointly through coupled matrix and tensor factorizations. Joint analysis of histology and in vitro samples enables us to pinpoint the discriminative features for healthy and cancerous state separation, e.g., for bone samples in our experiments. Collectively, our study aims to quantify three significant but traditionally qualitative structure-function relationships in multicellular organisms:

### 1. The relationship between relative cell positioning in a tissue and the functional state of that tissue

Epithelial tissues lose structural integrity when they are damaged, but recover it as healing progresses; epithelial tumors also pass through this “damaged” stage, and ultimately lose this integrity altogether. Discriminating between these not-quite-healthy conditions is one of the most problematic issues in pathology. A similar case occurs in bone tissues: a healing fracture (i.e., fracture callus) so closely resembles osteosarcoma in H&E sections that physicians amputate the limbs of children as a precautionary measure. Finally, the treatments for traumatic brain injury and brain tumors differ significantly, so early and accurate diagnosis would improve outcomes tremendously. Our approach places several numeric features on the healthy and cancerous states, thereby clarifying the most significant differences between them. Both local and global metrics in our cell graphs define these features.

### 2. The relative importance of cell clusters vs. single cells in the functional state of a tissue

Epithelial cells are literally bound together by a series of cell-cell junctions that permit them to exchange metabolites, restrict paracellular transport, and even distribute tensile and compressive forces across a group of cells, thereby reducing the damage in any single cell. Osteoblasts maintain close communication via canaliculi, and the importance of intercellular contact between neighboring neurons is unquestioned. Yet, all of these contacts are somewhat plastic in even healthy tissues. What types of changes in these connections are permitted in healthy tissues, and when are the changes so dramatic as to reflect a loss of functional communication? While the concepts of dynamics of cell-to-cell communication are widely accepted, we have yet to develop accurate ways of finding the most important signs of meaningful changes in this communication. Our local metrics (e.g., clustering coefficient, degree, etc.) are representations of this communication between individual cells, and our global metrics (e.g., number of connected components, percentage of end points, etc.) sample interactions between neighboring clusters of cells. The numeric values of these features collectively quantify the concept of cell-cell cooperativity.

### 3. The linkage between gene/protein expression and phenotype in cells and tissues

Reductionist methods have identified thousands of genes in the human genome, and microarray and proteomic methods can routinely quantify their relative expression state in cells and tissues. Yet, a fundamental question remains: what is the relationship between combinations of genes/proteins and a given phenotype in a tissue, or even a single cell? This question will remain unanswered until we develop quantitatively rigorous methods for defining phenotypic state, comparable to the molecular expression methods we now use. While we have yet to address this question directly, the work described in this manuscript provides a framework for tackling this question in the near future. By inducing a known change in gene expression in a cell or tissue and calculating the change in metrics that capture functional state, we will be better equipped to identify these relationships, which form the basis of all structure/function studies in multicellular organisms.

Even though joint analysis of in vitro and histology samples can improve our understanding, in this paper, we have also studied organizational limitations of our 3D cell cultures by comparing them with the actual tissues and demonstrated that there is a quantifiable structural difference between our in vitro and in vivo (histology) samples.

## Supporting Information

Table S1 Cell-graph features and their descriptions. (During the analyses in the Results section, in subsections 1, 2 and 3, three features, i.e., effective hop diameter, number of central points and percentage of central points, are excluded.)
(DOCX)
