# Supplementary material for: Coupled Analysis of In Vitro and Histology Tissue Samples to Quantify Structure-Function Relationship
Source: PLoS One. 2012 Mar 30;7(3):e32227. doi: 10.1371/journal.pone.0032227 (PMC3316529; doi:10.1371/journal.pone.0032227)
Supplement: Table S1 — Cell-graph features and their descriptions. (During the analyses in the Results section, in subsections 1, 2 and 3, three features, i.e., effective hop diameter, number of central points and percentage of central points, are excluded.) (DOCX) [file pone.0032227.s001.docx]

**Table S1.** Cell-graph features and their descriptions. (During the analyses in the Results section, in subsections 1, 2 and 3, three features, i.e., effective hop diameter, number of central points and percentage of central points, are excluded.)

| **Feature Label** | **Feature Descriptions** |
| --- | --- |
| Average Degree | Number of edges per node |
| Clustering Coefficient C | Ratio of total number of edges among the neighbors of the node to the total number of edges that can exist among the neighbors of the node per node |
| Clustering Coefficient D | Ratio of total number of edges among the neighbors of the node and the node itself to the total number of edges that can exist among the neighbors of the node and the node itself per node |
| Clustering Coefficient E | Ratio of total number of edges among the neighbors of the node to the total number of edges that can exist among the neighbors of the node per node excluding the isolated nodes |
| Average Eccentricity | Average of node eccentricities where the eccentricity of a node is the maximum shortest path length from the node to any other node in the graph. |
| Diameter | Maximum of node eccentricities. |
| Radius | Minimum of node eccentricities. |
| Average Eccentricity 90 | Average of node eccentricities to reach 90% of the nodes in the graph. |
| Diameter 90 | Maximum of node eccentricities to reach 90% of the nodes in the graph. |
| Radius 90 | Minimum of node eccentricities to reach 90% of the nodes in the graph. |
| Average Path Length | Average distance between the nodes of a graph, where the distance between two nodes is the number of edges in the shortest path that connects them. |
| Effective Hop Diameter | is , where hop plot exponent is the slope of the least squares estimated line fitted to the hop plot values in log–log domain, where the hop plot value for is the number of node pairs for which the path length between the pairs is less than or equal to , is the number of nodes, and is the number of edges in the graph. 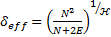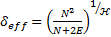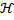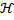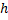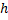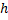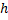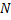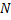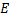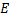 |
| Hop Plot Exponent | Slope of the line fitted to the hop plot values in log–log domain, where the hop plot value for hop *h* is the number of node pairs for which the path length between the pairs is less than or equal to *h* |
| Giant Connected Component Ratio | Ratio between the number of nodes in the largest connected component in the graph and total the number of nodes |
| Number of Connected Components | Number of clusters in the graph excluding the isolated nodes |
| Average Connected Component Size | Number of nodes per connected component |
| Percentage of Isolated Points | Percentage of the isolated nodes in the graph, where an isolated node has a degree of 0 |
| Percentage of End Points | Percentage of the isolated nodes in the graph, where an isolated node has a degree of 1 |
| Number of Central Points | Number of nodes within the graph whose eccentricity is equal to the graph radius |
| Percentage of Central Points | Percentage of the central points in the graph |
| Average of Edge Lengths (mean) | Statistics of the edge length distribution in the graph |
| Standard Deviation of Edge Lengths (std) |  |
| Skewness of Edge Lengths |  |
| Kurtosis of Edge Lengths |  |
| Number of nodes | Number of cells in the tissue sample |
| Number of edges | Number of pairwise relationships between the cells in the tissue sample. |
